# Influence of Baseline CT Body Composition Parameters on Survival in Patients with Pancreatic Adenocarcinoma

**DOI:** 10.3390/jcm11092356

**Published:** 2022-04-22

**Authors:** Nick Lasse Beetz, Dominik Geisel, Christoph Maier, Timo Alexander Auer, Seyd Shnayien, Thomas Malinka, Christopher Claudius Maximilian Neumann, Uwe Pelzer, Uli Fehrenbach

**Affiliations:** 1 Department of Radiology, Charité–Universitätsmedizin Berlin, Corporate Member of Freie Universität Berlin and Humboldt-Universität zu Berlin, 13353 Berlin, Germany; dominik.geisel@charite.de (D.G.); christoph.maier@charite.de (C.M.); timo-alexander.auer@charite.de (T.A.A.); seyd.shnayien@charite.de (S.S.); uli.fehrenbach@charite.de (U.F.); 2 Berlin Institute of Health, 10178 Berlin, Germany; 3 Department of Surgery, Charité–Universitätsmedizin Berlin, Corporate Member of Freie Universität Berlin and Humboldt-Universität zu Berlin, 13353 Berlin, Germany; thomas.malinka@charite.de; 4 Department of Oncology, Charité–Universitätsmedizin Berlin, Corporate Member of Freie Universität Berlin and Humboldt-Universität zu Berlin, 10117 Berlin, Germany; christopher.neumann@charite.de (C.C.M.N.); uwe.pelzer@charite.de (U.P.)

**Keywords:** pancreatic cancer, body composition, survival, computed tomography, CT, artificial intelligence, AI, surgery, oncology, imaging predictors

## Abstract

Pancreatic cancer is the seventh leading cause of cancer death in both sexes. The aim of this study is to analyze baseline CT body composition using artificial intelligence to identify possible imaging predictors of survival. We retrospectively included 103 patients. First, the presence of surgical treatment and cut-off values for sarcopenia and obesity served as independent variates. Second, the presence of surgery, subcutaneous adipose tissue (SAT), visceral adipose tissue (VAT), and skeletal muscle index (SMI) served as independent variates. Cox regression analysis was performed for 1-year, 2-year, and 3-year survival. Possible differences between patients undergoing surgical versus nonsurgical treatment were analyzed. Presence of surgery significantly predicted 1-year, 2-year, and 3-year survival (*p* = 0.01, <0.001, and <0.001, respectively). Across the follow-up periods of 1-year, 2-year, and 3-year survival, the presence of sarcopenia became an equally important predictor of survival (*p* = 0.25, 0.07, and <0.001, respectively). Additionally, increased VAT predicted 2-year and 3-year survival (*p* = 0.02 and 0.04, respectively). The impact of sarcopenia on 3-year survival was higher in the surgical treatment group (*p* = 0.02 and odds ratio = 2.57) compared with the nonsurgical treatment group (*p* = 0.04 and odds ratio = 1.92). Fittingly, a lower SMI significantly affected 3-year survival only in patients who underwent surgery (*p* = 0.02). Especially if surgery is performed, AI-derived sarcopenia and reduced muscle mass are unfavorable imaging predictors.

## 1. Introduction

Worldwide, exocrine pancreatic cancer is the seventh leading cause of cancer death in both sexes. Because of its poor prognosis, exocrine pancreatic cancer accounts for almost as many deaths as there are cases even if potentially curative surgery is performed [1]. Unfortunately, due to the late presentation of the disease, only 15 to 20 percent of patients are candidates for surgery. Systemic chemotherapy combinations such as 5-fluorouracil, folinic acid, irinotecan, and oxaliplatin (FOLFIRINOX) have been shown to achieve conversion to resectability in about a third of patients with locally advanced exocrine pancreatic cancer [2,3]. However, overall prognosis remains poor with a 5-year survival rate of approximately 10% [4].

By far the most common exocrine pancreatic cancer is pancreatic adenocarcinoma which accounts for about 85% of all pancreatic cancers [5]. Known risk factors for exocrine pancreatic cancer include obesity, diabetes mellitus, smoking, chronic pancreatitis, and a positive family history [6]. Age has been identified as another important risk factor for developing exocrine pancreatic cancer. In fact, 70% of patients are older than 65 [7]. Frailty is a common concern in these elderly patients: sarcopenia and cachexia are associated with perioperative complications, prolonged hospitalization, and poorer overall survival [8,9]. Therefore, early identification of sarcopenic patients at risk is needed.

Computed tomography (CT) is the preferred modality for initial imaging evaluation of patients with suspected pancreatic cancer and allows correct assessment of vascular infiltration [10,11]. As an alternative, magnetic resonance imaging (MRI) offers similar sensitivity and specificity in staging pancreatic cancer, but it is used less commonly because of its lower availability and higher cost [12,13].

Body composition describes the percentages of different body tissues in the human body and has long been used as a measure of physical fitness [14,15]. CT imaging datasets can be used for artificial intelligence (AI)-based body composition analysis, which automatically differentiates the relative proportions of various tissues using muscle and adipose tissue parameters including skeletal muscle index (SMI), visceral adipose tissue (VAT), and subcutaneous adipose tissue (SAT) [16]. Unlike the body mass index (BMI), this individual metabolic information can identify frail patients, for example, patients with sarcopenic obesity, who have a normal BMI with reduced muscle mass and severe obesity [17]. Additionally, body composition analysis can be used to detect sarcopenia, which is defined as the presence of low muscle mass using sex-specific cut-off values, and sarcopenic obesity, which is defined as the combined presence of both sarcopenia and obesity [18,19].

Body composition describes the percentages of different body tissues in the human body and has long been used as a measure of physical fitness [14,15]. CT imaging datasets can be used for artificial intelligence (AI)-based body composition analysis, which differentiates the relative proportions of various tissues. Muscle and adipose tissue parameters including visceral adipose tissue (VAT), and subcutaneous adipose tissue (SAT), and skeletal muscle index (SMI) are automatically calculated [16]. Unlike the body mass index (BMI), this individual metabolic information can detect frail patients who suffer from sarcopenia or sarcopenic obesity. Sarcopenia is defined as the presence of low muscle mass using sex-specific cut-off values, whereas sarcopenic obesity is defined as the combined presence of both sarcopenia and obesity [18,19]. For example, patients with severe obesity and reduced muscle mass may have a normal BMI but suffer from sarcopenic obesity [17].

Body composition parameters have been recognized as outcome predictors in many oncological diseases and cardiovascular conditions. For example, it has been reported that body composition predicts progression of aortic enlargement, coronary heart disease, and outcomes in patients undergoing transcatheter aortic valve implantation [20,21,22]. Moreover, in patients with esophageal cancer, severe complications, prolonged hospitalization, and overall survival are influenced by sarcopenia assessed by CT body composition analysis [23].

The hypothesis of this study is that baseline CT body composition parameters may influence survival in patients with pancreatic adenocarcinoma regardless of whether they undergo surgical or nonsurgical treatment. To test this hypothesis, we analyze AI-based body composition in CT scans obtained at baseline in a retrospective dataset to identify possible imaging predictors of 1-year, 2-year, and 3-year survival.

## 2. Materials and Methods

### 2.1. Study Design

In this single-center cohort study, we analyzed body composition in a retrospective dataset of patients with pancreatic adenocarcinoma who underwent baseline CT for initial evaluation. The study was performed in compliance with the Declaration of Helsinki and approved by the institutional review board.

### 2.2. Patient Population and Characteristics

**Figure 1:**
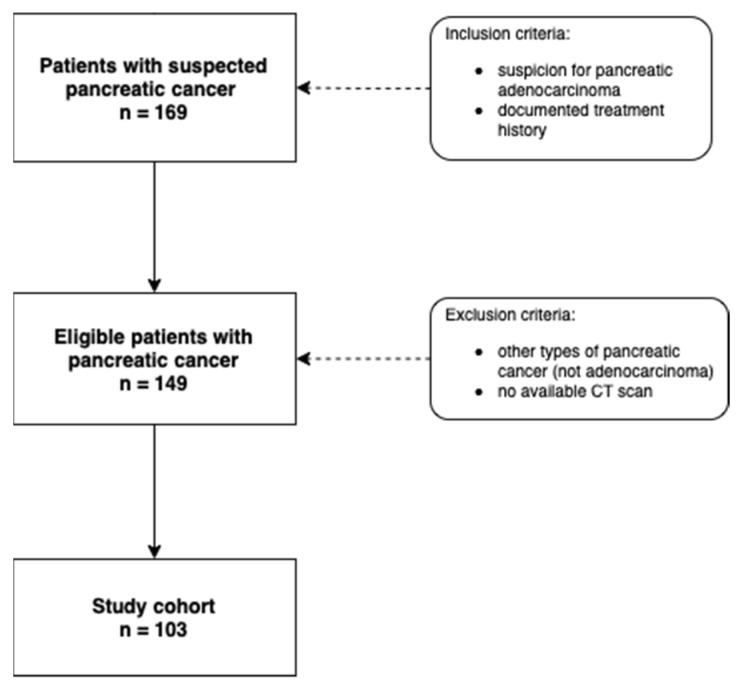
Flowchart depicting the inclusion and exclusion criteria for this study. CT = computed tomography, n = number.

All patients were diagnosed with pancreatic adenocarcinoma and underwent baseline CT imaging at the time of diagnosis. They were referred for CT from the Department of Surgery or the Department of Oncology. All patients included were diagnosed between 2010 and 2017. Only patients with a documented treatment history including survival data were included in this study. Patients with other types of pancreatic cancer and patients without CT scans for initial assessment were excluded. None of the patients included in this study had severe ascites. A flowchart depicting the inclusion and exclusion criteria is shown in Figure 1.

### 2.3. Data Collection, Follow-Up, and Endpoints

In total, 1-year, 2-year, and 3-year survival after baseline CT imaging were defined as endpoints. All data were retrieved from the clinical database and patient records. The patients diagnosed with pancreatic adenocarcinoma underwent treatment at our university center and were followed up for 5 years. Follow-up rates were 99% at 1 year (1 patient lost to follow-up), 94% at 3 years (6 patients lost), and 89% at 5 years (11 patients lost).

### 2.4. AI-Based Body Composition Analysis

**Figure 2:**
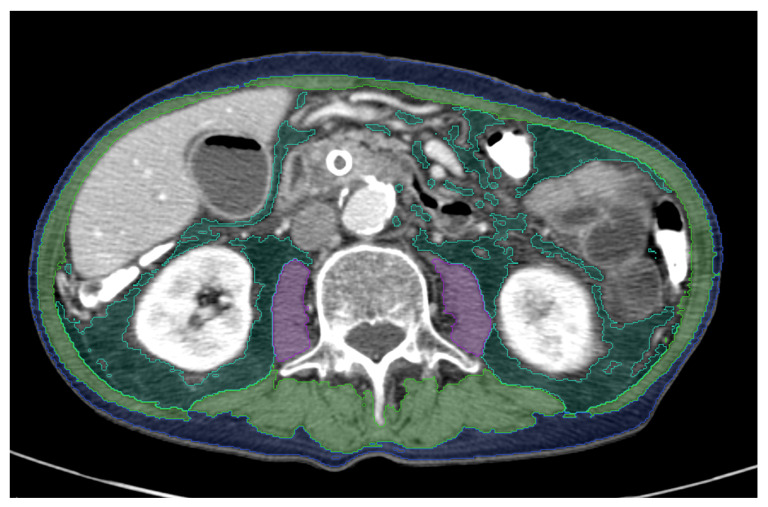
Example illustrating the result of the PACS-integrated AI-based body composition analysis in a patient with pancreatic adenocarcinoma. The patient has reduced muscle mass with a SMI of 28.2 cm^2^/m^2^ indicating the presence of sarcopenia. There is accumulation of gas in the gallbladder caused by a common bile duct stent. Each segmented tissue is coded with a different color: psoas muscle = purple, skeletal muscle = green, SMI = skeletal muscle index, visceral fat = dark green, subcutaneous fat = blue. Tissue areas were automatically calculated.

Analysis of body composition was performed on available baseline CT datasets acquired before surgical or non-surgical treatment at the Department of Radiology and at external locations. We used a picture archiving and communications system (PACS)-integrated AI-based software tool (Visage version 7.1, Visage Imaging GmbH, Berlin, Germany) which is based on a convolutional neural network. The network consists of nine blocks: four upsampling blocks, four downsampling blocks, and one in between. The initial training data consisted of 200 axial CT images of the third lumbar vertebra (L3) level, which were acquired at internal and at external locations with various CT protocols. Skeletal muscle, psoas muscle, VAT, and SAT were automatically separated. Each tissue class was coded with a different color. Automatic segmentation was checked by an experienced radiologist. AI-based image segmentation was manually corrected in few cases to avoid false area calculation, for example, when hypodense stool in the intestine was misinterpreted as body fat. For each tissue class the software tool automatically calculated the area in square centimeters (cm^2^) and density in Hounsfield units [16]. The skeletal muscle index (SMI) was calculated using the following formula: skeletal muscle area including the psoas muscle (cm^2^)/body surface area (m^2^). For internal and external validation of the AI software tool its performance has already been compared with that of an established semi-automatic segmentation tool regarding speed and accuracy of tissue area calculation. The established workflow is integrated in a widely used PACS [16]. An example of AI-based automated analysis of body composition derived at the L3 level is shown in Figure 2.

### 2.5. Statistical Analysis

For analysis of 1-year, 2-year, and 3-year survival, multivariate Cox proportional hazard regression analysis was performed, and a *p*-value ≤ 0.05 was considered to indicate a significant difference. All data analyses were performed using IBM SPSS Statistics version 27 (International Business Machines Corporation, IBM). The cut-off for sarcopenia was defined as SMI ≤ 38.5 cm^2^/m^2^ in women and SMI ≤ 52.4 cm^2^/m^2^ in men. The cut-off for obesity was defined as a BMI ≥ 30. For each outcome endpoint, including 1-year, 2-year, and 3-year survival, the dependent variant was defined as death of the patient. First, presence of surgical treatment and cut-off values for sex-specific sarcopenia and obesity served as independent variates. Second, the presence of surgery, SMI, VAT, and SAT served as independent variates. Different chemotherapy regimens (Gemcitabine, Gemcitabine + nab-paclitaxel, or FOLFIRINOX), BMI, age, and sex as possible confounders were also included in the multivariate analysis. Except for presence of surgery, the same independent variates were used for comparison of the surgical and nonsurgical treatment groups. Proportional hazard assumption was tested. Kaplan–Meier curves were plotted for 3-year survival, and log-rank testing was performed.

## 3. Results

### 3.1. Baseline Data

A total of 103 patients with a mean age of 62 ± 11 years at the time of diagnosis (ranging from 37 to 84 years) were included in this study. There were 41 women and 62 men. Mean weight was 76 ± 14 kg, and mean height was 171 ± 9 cm. BMI was calculated using the following formula: BMI = weight/height^2^ (kg/m^2^). Mean BMI was 26 ± 5. All patients underwent chemotherapy: 45 were treated with gemcitabine, 43 were treated with gemcitabine + nab-paclitaxel, and 15 with FOLFIRINOX. Additionally, 46 had surgery (pylorus preserving pancreatoduodenectomy), whereas 57 patients underwent nonsurgical treatment only. Further clinical characteristics are compiled in Table 1.

### 3.2. AI-Based Body Composition Parameters and Cut-Off Values

All AI-based body composition parameters were derived at the third lumbar vertebra level (L3). The mean SMI was 45 ± 9 cm^2^/m^2^. Mean VAT was 112 ± 82 mm^2^, and mean SAT 159 ± 82 mm^2^. In accordance with the guideline of the European Working Group on Sarcopenia, the cut-off for sarcopenia was defined as SMI ≤ 38.5 cm^2^/m^2^ in females and SMI ≤ 52.4 cm^2^/m^2^ in males, whereas obesity was defined as a BMI ≥ 30 [18,19]. In total, 65 patients (63%) had sarcopenia, 21 patients had obesity (21%), and 8 patients (8%) had sarcopenic obesity. All results are compiled in Table 2.

### 3.3. Cox Regression Survival Analysis Using AI-Derived Body Composition Parameters as Independent Variates

In total, 1-year, 2-year, and 3-year survival was predicted significantly by the presence of surgery (*p* = 0.01, <0.001, and <0.001, respectively). Across the follow-up periods of 1 year, 2 years, and 3 years, the presence of sarcopenia became an equally important predictor of survival (*p* = 0.25, 0.07, and <0.001, respectively). Additionally, increased VAT predicted 2-year and 3-year survival (*p* = 0.01 and 0.04, respectively). The impact of sarcopenia on 3-year survival was higher in the surgical treatment group (*p* = 0.02 and odds ratio = 2.57) than in the nonsurgical treatment group (*p* = 0.04 and odds ratio = 1.92). Fittingly, a lower SMI significantly influenced 3-year survival only in patients who underwent surgery (*p* = 0.02). 1-year, 2-year, and 3-year survival were not significantly predicted by any other independent variate including obesity, sex, age, BMI, and SAT. The different chemotherapy regimens did not significantly influence the results. All results are compiled in Table 3 and Table 4.

**Figure 3:**
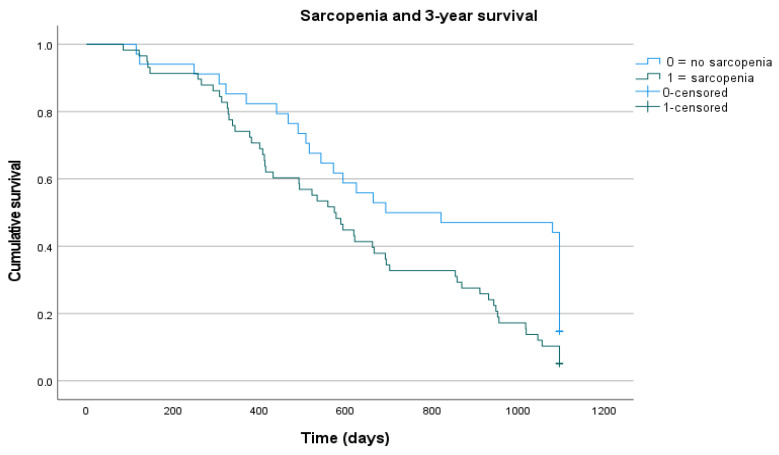
Kaplan-Meier curve demonstrating that over the total follow-up period of three years the AI-derived body composition parameter sarcopenia evolves as a significant imaging predictor of survival in patients with pancreatic adenocarcinoma. Patients suffering from sarcopenia had significantly poorer survival rates (log-rank, *p* = 0.006).

Additionally, a Kaplan–Meier curve demonstrates that, over the total follow-up period of three years, the AI-derived body composition parameter sarcopenia evolves as a significant imaging predictor of survival in patients with pancreatic adenocarcinoma (Figure 3). Patients suffering from sarcopenia had significantly poorer survival rates (log-rank, *p* = 0.006).

### 3.4. Comparison of Effects of AI-Based Body Composition Parameters on Survival between Patients Undergoing Surgical Versus Nonsurgical Treatment

In patients with pancreatic adenocarcinoma whose treatment included surgery, 3-year survival was predicted significantly by the following AI-based body composition parameters: presence of sarcopenia (*p* = 0.02), reduced SMI (*p* = 0.02), and increased VAT (*p* = 0.01). Conversely, 3-year survival was not significantly influenced by any other independent variate including obesity (*p* = 0.18), sex (*p* = 0.39), age (*p* = 0.32), BMI (*p* = 0.25), and SAT (0.38). The different chemotherapy regimens did not significantly influence survival.

In patients with pancreatic adenocarcinoma undergoing nonsurgical treatment only, 3-year survival was predicted significantly by the AI-based body composition parameter presence of sarcopenia (*p* = 0.04). 3-year survival was not significantly influenced by any other independent variate including obesity (*p* = 0.65), sex (*p* = 0.44), age (*p* = 0.94), and BMI (*p* = 0.96), or by the AI-based body composition parameters SMI (*p* = 0.38), VAT (*p* = 0.35), and VAT (*p* = 0.62). The different chemotherapy regimens did not significantly influence survival.

Finally, the impact of sarcopenia on 3-year survival was higher in the surgical treatment group (odds ratio = 2.57) than in the nonsurgical treatment group (odds ratio = 1.92). For all imaging survival predictors, the Proportional Harzard assumption was satisfied. All results are compiled in Table 5.

## 4. Discussion

In this retrospective study, we analyzed the role of artificial intelligence-based body composition analysis obtained at baseline CT in predicting outcome of patients with pancreatic adenocarcinoma. Surgery was found to significantly predict 1-year, 2-year, and 3-year survival, whereas sarcopenia emerged as an equally significant predictor of survival over the total follow-up period. Moreover, increased VAT at L3 level significantly influenced 2-year and 3-year survival. Comparison of the effects of AI-based body composition parameters on survival between treatment groups showed that the impact of sarcopenia on 3-year survival was higher in patients whose treatment included surgery compared with patients undergoing nonsurgical treatment only. Fittingly, a lower SMI as an indicator of a small skeletal muscle mass significantly predicted 3-year survival only in patients who underwent surgery.

Assessment of sarcopenia in patients with pancreatic cancer is important as it is associated with poorer survival, higher toxicity of chemotherapy, and more perioperative complications [24,25]. Unfortunately, anthropometric measures such as BMI and waist circumference are only useful for initial assessment of obesity [26]. Conventional methods to determine a patient’s body composition include bioelectrical impedance analysis, which estimates the percentage of body fat from water impedance, and dual-energy x-ray absorptiometry (DEXA), which uses a very low dose of radiation and provides accurate estimates of body fat percentage [27,28,29]. However, modern imaging techniques including CT and MRI allow straightforward analysis of body composition and quantification of sarcopenia from a single axial image acquired at the third lumbar vertebra. AI-based body composition analysis does not require additional radiation dose or examination time for the patient [30,31]. Recent studies indicate that CT body composition analysis has evolved as an objective measurement of a patient’s physical fitness [32,33]. Similar to other studies investigating sarcopenia in pancreatic cancer [34], we found that 65% of the patients included in our study suffered from sarcopenia. This worryingly high proportion of sarcopenic patients underlines the importance of body composition analysis. Moreover, CT scans can be used to measure Hounsfield Unit Average Calculation (HUAC) which has been shown to be a predictive marker of muscle density and fatty infiltration [35,36,37].

Other studies have already identified reduced skeletal muscle mass as an independent prognostic factor. For example, Ninomiya et al. have shown that sarcopenic patients with resectable pancreatic cancer have poorer outcome, whereas Choi et al. have demonstrated that sarcopenia is associated with lower survival rates in locally advanced and metastatic pancreatic cancer [38,39]. Interestingly, our data show that the impact of sarcopenia is higher in patients who undergo curative intended surgery compared with patients without surgical treatment. This can be explained by the fact that sarcopenia is a risk factor for postoperative complications, which may reduce overall survival [40]. Nutritional modification and exercise training have been shown to improve sarcopenia and may therefore be recommended in sarcopenic patients identified by AI-based body composition analysis [41,42].

Another important component of individual body composition is the amount of visceral fat or VAT, which is increased in obese patients. The role of VAT in patients with pancreatic cancer is controversial. Hsu et al. have demonstrated that VAT does not influence overall survival in pancreatic cancer [43]. In contrast, our results suggest that increased VAT is a negative imaging predictor of survival, which can be explained by the fact that obesity is closely connected with muscle atrophy and altered muscle protein synthesis. This reciprocal regulation by adipose tissue and skeletal muscle dysfunction is the reason why many elderly patients suffer from both sarcopenia and obesity [44,45]. Furthermore, obesity is associated with many cardiovascular diseases, which may also contribute to overall patient survival [46].

The influence of body composition on various clinical outcomes has been broadly studied, and body composition has thus become an accepted risk factor. As this valuable metabolic information can easily be extracted from CT images, there is a growing demand for automatic software solutions. In this study, we for the first time use an already established fully automatic PACS-integrated AI-based software solution for survival analysis of cancer patients. Individual body composition analysis can be performed without transfer of any critical patient data to external software, and the additional metabolic information can be extracted without the need for further examination and be added to the imaging report by the radiologist.

For the first time, this study uses a PACS-integrated AI-based body composition analysis tool to predict survival in patients with pancreatic adenocarcinoma. Besides the known influence of sarcopenia on survival, our results provide evidence that increased visceral fat is a negative predictor of survival. Moreover, we show that sarcopenia has a greater impact in patients who undergo surgery compared with patients undergoing nonsurgical treatment only. We believe that in the future imaging predictors from routine CT might be used to prevent negative clinical outcomes, e.g., by providing adequate protein diet and exercise training in sarcopenic patients. These treatment options might improve patient’s physiological fitness for surgery and chemotherapy, and reduce treatment-associated complications.

Our study is limited by the use of a retrospective dataset. The study design prohibits any valid conclusion to be drawn regarding a potential causal relationship between tumor burden and sarcopenia. Even though multiple retrospective studies have shown the usefulness of imaging-based body composition analysis, this study does not provide an evaluation of the patient´s functional status such as grip strength measurements. Even though our study population is comparatively large, the results should be compared with conventional parameters including body impedance and be confirmed in a prospective trial. Tumor burden was not determined in this study cohort and could have also affected outcomes. Finally, as many patients are referred to our interdisciplinary cancer center for evaluation of surgical treatment options, there might be a selection bias towards a healthier study population.

## 5. Conclusions

AI-based body composition analysis in pancreatic adenocarcinoma provides important metabolic information for predicting patient survival. Especially if surgery is performed the presence of sarcopenia and a reduced muscle mass are unfavorable imaging predictors and should warrant additional special care to improve outcome. Moreover, a higher amount of visceral fat appears to be associated with poorer survival.

## Figures and Tables

**Table 1:** Clinical characteristics of all patients with pancreatic adenocarcinoma included in our retrospective analysis. BMI = body mass index. FOLFIRINOX = 5-fluorouracil, folinic acid, irinotecan, and oxaliplatin. PPPD = pylorus preserving pancreatoduodenectomy. * Median ± standard deviation.

	Total (n = 103)
Age, years *	62 ± 11
Sex, n (%)	
female	41 (40%)
male	62 (60%)
BMI *	26 ± 5
Chemotherapy, n (%)	
Gemcitabine	45 (44%)
Gemcitabine + nab-paclitaxel	43 (42%)
FOLFIRINOX	15 (15%)
First-line treatment, n (%)	
Surgical (PPPD)	46 (45%)
Nonsurgical	57 (55%)

**Table 2:** AI-derived body composition parameters at the third lumbar vertebra level in patients with pancreatic adenocarcinoma. SMI = skeletal muscle index. VAT = visceral adipose tissue. SAT = subcutaneous adipose tissue. * Median ± standard deviation.

Body Composition Parameter	Value
SMI (cm^2^/m^2^) *	45 ± 9
VAT (mm^2^) *	112 ± 82
SAT (mm^2^) *	159 ± 82
Sarcopenia	65 (63%)
Obesity	21 (20%)
Sarcopenic obesity	8 (8%)

**Table 3:** Cox regression analysis of all patients with pancreatic adenocarcinoma. Presence of surgery (pylorus preserving pancreatoduodenectomy), chemotherapy, and AI-derived cut-off values for sarcopenia and obesity served as independent variates. AI = artificial intelligence, CI = confidence interval.

	1-Year Survival		2-Year Survival		3-Year Survival	
Variate	*p*-value	Odds Ratio (CI)	*p*-value	Odds Ratio (CI)	*p*-value	Odds Ratio (CI)
Surgery	**0.01**	0.25 (0.08–0.74)	**<0.001**	0.28 (0.16–0.51)	**<0.001**	0.45 (0.29–0.70)
Chemotherapy	0.34	1.35 (0.73–2.49)	0.22	1.25 (0.88–1.77)	0.32	1.16 (0.86–1.56)
Sarcopenia	0.25	1.84 (0.65–5.17)	0.07	1.72 (0.95–3.12)	**<0.001**	2.12 (1.30–3.46)
Obesity	0.54	0.67 (0.19–2.36)	0.94	0.97 (0.50–1.88)	0.78	1.08 (0.63–1.85)
Total number	103		103		103	
Lost to follow-up	1 (1%)		6 (6%)		11 (11%)	

**Table 4:** Cox regression analysis of all patients with pancreatic adenocarcinoma. Surgery, sex, age, BMI, chemotherapy, and the AI-derived body composition parameters SMI, VAT, and SAT served as independent variates.

	1-Year Survival		2-Year Survival		3-Year Survival	
Variate	*p*-value	Odds Ratio (CI)	*p*-value	Odds Ratio (CI)	*p*-value	Odds Ratio (CI)
Sex	0.26	1.80 (0.65–4.96)	0.47	1.27 (0.67–2.41)	0.19	1.44 (0.83–2.51)
Age	0.17	1.03 (0.99–1.08)	0.25	1.02 (0.99–1.05)	0.49	1.01 (0.98–1.03)
Chemotherapy	0.45	1.29 (0.66–2.53)	0.39	1.18 (0.81–1.73)	0.80	1.04 (0.76–1.43)
Surgery	**0.02**	0.28 (0.09–0.85)	**<0.01**	0.32 (0.17–0.58)	**0.01**	0.52 (0.32–0.83)
BMI	0.62	1.00 (0.99–1.01)	0.80	1.00 (0.99–1.01)	0.43	1.00 (1.00–1.01)
SMI	0.85	1.00 (0.99–1.01)	0.39	1.00 (0.99–1.00)	0.08	1.00 (0.99–1.00)
VAT	0.86	1.00 (1.00–1.00)	**0.01**	1.00 (1.00–1.00)	**0.04**	1.00 (1.00–1.00)
SAT	0.20	1.00 (1.00–1.00)	0.41	1.00 (1.00–1.00)	0.32	1.00 (1.00–1.00)
Total number	103		103		103	
Lost to follow-up	1 (1%)		6 (6%)		11 (11%)	

**Table 5:** Different effects of AI-based body composition parameters on 3-year survival in patients undergoing surgical treatment and in patients undergoing nonsurgical treatment.

(a) Use of chemotherapy and AI-derived cut-off values for sarcopenia and obesity as independent variates. AI = artificial intelligence, CI = confidence interval.				
	**3-Year Survival**			
	**Nonsurgical treatment**		**Surgical treatment**	
Variate	*p*-value	Odds ratio (CI)	*p*-value	Odds ratio (CI)
Chemotherapy	0.24	1.24 (0.87–1.77)	0.77	0.92 (0.53–1.60)
Sarcopenia	**0.04**	1.92 (1.02–3.62)	**0.02**	2.57 (1.13–5.82)
Obesity	0.65	0.85 (0.43–1.70)	0.18	1.83 (0.76–4.42)
Total number	57		46	
Lost to follow-up	6 (11%)		5 (11%)	
(b) Use of sex, age, BMI, and the AI-derived body composition parameters SMI, VAT, and SAT as independent variates.				
	**3-Year Survival**			
	**Nonsurgical treatment**		**Surgical treatment**	
Variate	*p*-value	Odds ratio (CI)	*p*-value	Odds ratio (CI)
Sex	0.44	1.36 (0.62–2.99)	0.39	1.43 (0.64–3.19)
Age	0.94	1.00 (0.97–1.03)	0.32	1.02 (0.98–1.06)
Chemotherapy	0.39	1.18 (0.81–1.74)	0.37	0.73 (0.36–1.45)
BMI	0.96	1.00 (0.99–1.01)	0.25	1.01 (1.00–1.02)
SMI	0.38	1.00 (0.99–1.00)	**0.02**	0.99 (0.99–1.00)
VAT	0.35	1.00 (1.00–1.00)	**0.01**	1.00 (1.00–1.00)
SAT	0.62	1.00 (1.00–1.00)	0.38	1.00 (1.00–1.00)
Total number	57		46	
Lost to follow-up	6 (11%)		5 (11%)	

## Data Availability

The data presented in this study are available on request from the corresponding author.
